# Diagnostic accuracy of the InBiOS AMD rapid diagnostic test for the detection of *Burkholderia pseudomallei* antigen in grown blood culture broth

**DOI:** 10.1007/s10096-018-3237-3

**Published:** 2018-03-28

**Authors:** Marjan Peeters, Panha Chung, Hua Lin, Kristien Mortelmans, Chhundy Phe, Chentha San, Laura Maria Francisca Kuijpers, Syna Teav, Thong Phe, Jan Jacobs

**Affiliations:** 1 Department of Clinical Sciences, Institute of Tropical Medicine, Antwerp, Belgium; 2 Sihanouk Hospital Center of HOPE, Phnom Penh, Cambodia; 3 SRI International, Menlo Park, CA USA; 4 Department of Microbiology and Immunology, KU Leuven, Leuven, Belgium

**Keywords:** *Burkholderia pseudomallei*, Melioidosis, Rapid diagnostic test, Low-resource setting

## Abstract

To assess the diagnostic and operational performance of the InBiOS AMD rapid diagnostic test (RDT) (Seattle, USA) for the detection of *B. pseudomallei* in grown blood culture broth. The InBiOS RDT is a lateral flow immunoassay in a strip format detecting *B. pseudomallei* capsular polysaccharide in culture fluids, marketed for research only. Broth of blood culture bottles (BacT/Alert, bioMérieux, Marcy L’Etoile, France) sampled in adult patients at the Sihanouk Hospital Center of HOPE, Phnom Penh, Cambodia, during 2010–2017 and stored at − 80 °C was tested. They included samples grown with *B. pseudomallei* (*n* = 114), samples with no growth (*n* = 12), and samples with growth of other pathogens (*n* = 139, among which *Burkholderia cepacia* (*n* = 5)). Diagnostic sensitivity and specificity were 96.5% [95% confidence interval (CI): 91.3–98.6%] and 100% [CI: 97.5–100%] respectively. Background clearance and line intensities were good and very good. The RDT’s test strip, not housed in a cassette, caused difficulties in manipulation and biosafety. The centrifugation step prescribed by the procedure challenged biosafety, but processing of 19 *B. pseudomallei* samples without centrifugation showed similar results for line intensity and background clearance, compared to centrifugation. The InBiOS RDT showed excellent accuracy for detection of *B. pseudomallei* in grown blood culture broth. Provided operational adaptations such as cassette housing, it has the potential to reduce time to diagnosis of melioidosis.

**Electronic supplementary material:**

The online version of this article (10.1007/s10096-018-3237-3) contains supplementary material, which is available to authorized users.

## Introduction

Melioidosis is a community-acquired disease caused by the Gram-negative environmental bacterium *Burkholderia pseudomallei* [1, 2]. This disease, highly endemic in South-East Asia and northern Australia, has spread globally over the last century due to movement of people and cargo [3]. The global burden of the disease has recently been estimated to be 165,000 cases/year, of which approximately 89,000 (36000–227,000) succumb to the disease [4], with highest mortality rates recorded in low-resource settings [5, 6]. The most common disease manifestation is pneumonia, but a spectrum of clinical manifestations is possible, ranging from localized skin infections to fulminant sepsis [7]. About 44–67% of melioidosis cases present with sepsis [8–10], which is a major determinant of case fatality (up to 54%, [9]). One half of the deaths caused by melioidosis occur in the first 48 h after presentation to the hospital [11].

Diagnosis of melioidosis is currently done by microbiological culture of blood or other affected body specimens, which requires considerable laboratory infrastructure as well as trained staff and takes several days before completion [12].

Recently, InBiOS International Inc. (Seattle, WA, USA) released a rapid diagnostic test (RDT), the InBiOS Active Melioidosis Detect (AMD) RDT that detects *B. pseudomalle*i and *Burkholderia mallei* (*B. mallei*) capsular polysaccharide (CPS) in clinical specimens such as blood, urine, and pus [13]. When applied to grown blood culture broth, this test might shorten the time to diagnosis by 24 to 48 h in low-resource settings.

The present study aimed to assess the diagnostic accuracy of the InBiOS AMD RDT on grown blood culture broth sampled during routine patient care in a melioidosis-endemic setting and stored at − 80 °C. The study was conducted after the InBiOS AMD RDT had been assessed in a successful study pilot phase [14].

## Methods

### Study site

Sihanouk Hospital Center of HOPE is a 30-bed non-governmental organization hospital for adults providing affordable care for the poor, located in Phnom Penh, Cambodia, a country endemic for melioidosis [6, 15]. In 2016, care was given to 30,500 outpatients and 800 hospitalized patients. Since 2007, microbiological surveillance is conducted by consecutive blood cultures drawn in patients presenting with presumed bloodstream infection.

### Study design

This study was a retrospective evaluation of the InBiOS AMD RDT for detection of *B. pseudomallei* in stored blood culture broth. The performance of the InBiOS AMD RDT was first assessed in a pilot phase (SHCH, October 2016) and next in an extended evaluation phase (SHCH and ITM, March 2017). In January 2018, two additional studies were performed in SHCH to assess biosafety of the procedure: a first study to compare the test procedure with and without centrifugation and a second study to assess growth of *B. pseudomallei* on MacConkey agar incubated with processed test strips. The reference method was standard microbiological work-up of blood cultures [7]. Reporting of the methods and results was done according to the STARD guidelines for diagnostic studies (Supplementary file 3) [16].

### Blood culture methods and processing

Blood cultures, consisting of 2 × 10 ml sampled from separate venipunctures in paired aerobic (FA FAN, REF 259791) and anaerobic (FN FAN, REF 252793) BacT/Alert bottles (bioMérieux, Marcy L’Etoile, France), were processed with conventional methods as described previously [6]. From January 2016 onwards, the new bioMérieux blood culture bottles (paired aerobic and anaerobic) containing resins instead of charcoal were used (REF 410851 and REF 410852). *B. pseudomallei* was identified by the appearance of small, bipolar Gram-negative bacilli on Gram stain, growth as non-fermentative Gram-negative rods with wrinkled colony aspect on 5% sheep blood agar, positive oxidase reaction and resistance to colistin and gentamicin with susceptibility to amoxicillin-clavulanic acid [17]. Identification was confirmed using the API 20NE system (bioMérieux, Marcy L’Etoile, France) and if available, latex agglutination [18, 19]. Of each grown bottle pair, a 1.5 ml aliquot of blood culture broth was transferred into a cryovial and stored at − 80 °C. Bacterial isolates recovered from blood cultures were stored at − 80 °C on porous beads in cryopreservative (Microbank, Pro-Lab Diagnostics, Richmond Hill, Canada).

### The InBiOS AMD rapid diagnostic test

**Fig. 1:**
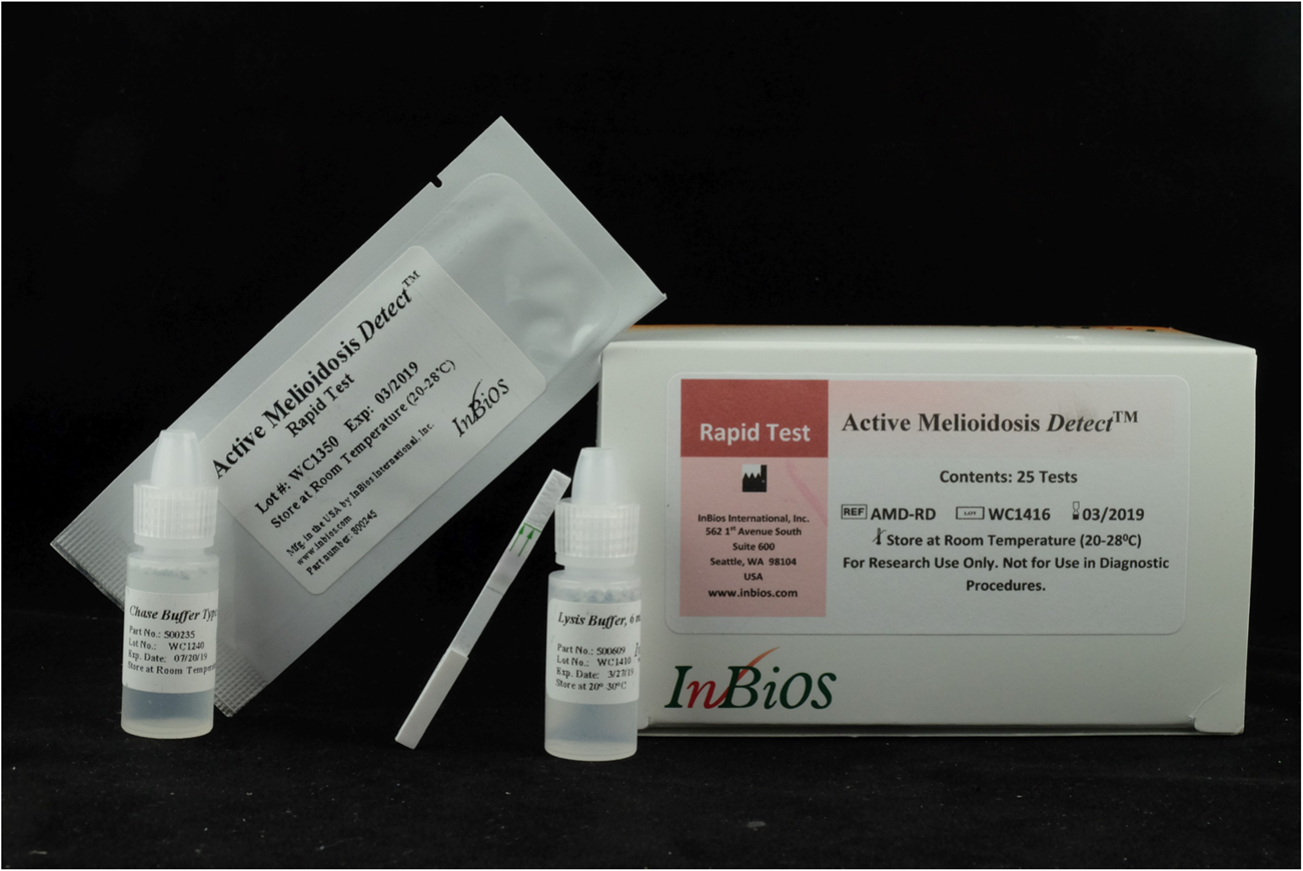
Package, RDT strip, and buffer vials provided

The InBiOS AMD RDT (InBiOS AMD Active Melioidosis *Detect*™, *ref*.: AMD-RD) is a lateral flow immunoassay in the form of a nitrocellulose membrane strip coated with a monoclonal antibody (mAB 3C5) to CPS produced by *B. pseudomallei* during infection. The RDT allows detection of the antigen in blood, serum, urine, pus, sputum, and culture fluids. According to the instructions for use (IFU), CPS of the closely related *B. mallei* is also detected [20]. The strips are individually packaged, and both a lysis and chase buffer are provided in each box of 25 strips (Fig. 1). A centrifugation step is required to perform the test on blood culture broth. The product is currently marketed for research only.

### Sample size calculation

The sample size of the extended evaluation study was calculated for an estimated sensitivity of 95%, with a desired precision of ± 5%, according to the DEEP guidelines [21]. Only the first sample of each patient was included. The minimal calculated sample size needed was 73 samples for both the *B. pseudomallei* panel and the competing panel. The competing panel was next extended to accommodate (i) regular pathogens and contaminants recovered from blood cultures in SHCH [6], as well as (ii) non-fermentative Gram-negative rods such as *Burkholderia cepacia* (*B. cepacia*) and *Pseudomonas* species. In addition, samples of blood cultures with no growth were added.

### Test procedure

**Fig. 2:**
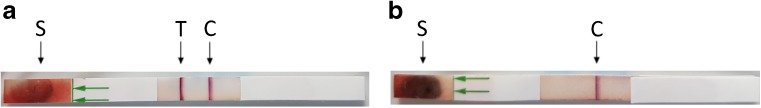
Definition of a positive and a negative reading of the InBiOS AMD RDT. **a** InBiOS AMD RDT positive for *B. pseudomallei* and **b** InBiOS AMD RDT negative for *B. pseudomallei*. Black arrows indicate (S) sample pad, (T) test line, and (C) control line

The InBiOS AMD RDTs were performed at room temperature according to the IFU. Apart from labeling of empty tubes and centrifugation of the samples, all steps were performed in a class II biosafety cabinet. Two sterile tubes for each sample—not supplied with the test kit—were labeled and in one of the tubes, two drops of lysis buffer was added. The cryovial containing 1.5 ml of blood culture broth was centrifuged at 3200×*g* for 10 min. The supernatants obtained were separated in the first tube, and the pellet obtained was suspended with the lysis buffer in the second tube. Immediately afterwards, 20 μl of this suspension was applied to the sample pad indicated by the arrows at the proximal end of the lateral flow strip (Fig. 2). The strips were placed on paper forms on which the sample identification had been written. Next, three drops of chase buffer were added to the inoculated sample pad and a timer was set to read the results after 15 min. The two readers were blinded to each other’s recordings and to the blood culture result. In case of discordant readings concerning the test result or the line intensity, a consensus was sought between the readers. Immediately after reading of the results, a picture was taken. Tests that scored negative were read again after 60 min to assess for delayed appearance of test lines.

### Definitions

A positive, valid test result was indicated by the appearance of both a control and test line (Fig. 2a). A negative, valid test result was indicated by the sole presence of a control line (Fig. 2b). In absence of a control line, the test was defined as invalid. Test line intensities were scored as “faint” (hardly visible) and “weak,” “medium,” or “strong” if the test line intensity was respectively weaker, equal, or stronger compared to the control line [22]. In addition, the strip background at the moment of reading was scored and recorded as “light” (optimal clearance), “medium” (background present but control line clearly visible), and “high” (background present, control line not clearly visible). In case of false-positive or false-negative results, the laboratory logbook was assessed to exclude clerical errors, the blood culture broth was inoculated onto sheep blood agar and incubated for 48 h, and the corresponding isolate was retrieved from storage and re-identified by standard biochemical methods. If this resulted in an identification different from the one recorded in the SHCH blood culture logbook, or if no isolate could be retrieved from the broth, the sample was excluded from subsequent analysis since the presence of *B. pseudomallei* in the blood culture broth sample could not be proven.

### Repeatability

Repeatability was assessed on 48 samples, randomly selected within each sample panel (23 samples of the competing panel, 6 samples with no growth, and 19 samples of the *B. pseudomallei* panel). For these 48 samples, broth was applied to two separate RDTs at the same time and by the same technician. Results were recorded as described above for both tests.

### Assessment of the prozone effect

Samples from the *B. pseudomallei* panel that showed negative or faint results were diluted in 0.9% sodium chloride (NaCl) and retested to exclude or demonstrate a prozone effect (i.e., the appearance of a false-low or a false-negative test line due to the excess of antigens in the sample). A prozone effect was defined as a sample that presented no test line or a faint test line when tested without dilution and a higher intensity of the test line upon dilution as recorded by two blinded observers [23].

### Procedure performed with and without centrifugation step

As the centrifuge step prescribed by the IFU may challenge biosafety, the procedure was performed in parallel with and without centrifugation on 19 grown blood culture broth samples. To perform the procedure without centrifugation, 20 μl of blood culture broth-lysis suspension was added to the test strip without centrifugation. Results were read and recorded after 15 min by two readers who were blinded to each other’s recordings.

### Test of the processed InBiOS strips for presence of viable bacteria

To assess biosafety of the InBiOS RDT, the InBiOS test strips used for the assessment of the procedure with and without centrifugation were placed on a MacConkey agar, incubated for 48 h at 35 °C and checked for growth. Growth on the MacConkey agar was identified according to standard procedures.

### Evaluation of the instructions for use and ease of use

The information mentioned in the IFU of the InBiOS AMD RDT product was assessed using a generic template for IFUs (Supplementary Table 2) [24]. In addition, ease of use and applicability of the InBiOS AMD RDT in the study setting were assessed and discussed among the observers during the laboratory testing.

### Database registration and statistical analysis

All results were first recorded on a paper form and afterwards transcribed into an electronic database (Microsoft Excel, Supplementary Table 1). Only the principal investigator and staff reading and recording the test results had access to this database. Sensitivity and specificity of the InBiOS AMD RDT compared to the identification of the blood culture isolate as the reference method test were calculated with 95% confidence intervals (CI) and expressed as consensus results at the first testing (results of repeatability testing were not considered). Inter-observer agreements for test line intensities and positive and negative test results were expressed by the percentage of overall agreement and by Kappa values. A Kappa value of 0.6–0.8 was considered good, and > 0.8 was considered excellent [22]. Differences in proportions were assessed using chi-square analysis. When the sample size was too small or when dependent samples were compared, respectively, Fisher exact tests or McNemar tests were used. The statistical analysis was performed with GraphPad software (www.graphpad.com) and Vassarstats (www.vassarstats.net). The clearance of the test background was expressed as the proportion of samples with light, medium, and high background, observed by the first reader. All data generated or analyzed during this study are included in this published article and its supplementary information files.

### Ethical approval

Ethical approval for the collection of blood culture broth and use of stored blood culture broth in diagnostic studies for infectious diseases was granted as part of the surveillance protocol: “Surveillance of antimicrobial resistance among consecutive blood culture isolates in tropical settings, V3.0” (IRB ITM 613/08, NECHR original protocol 009 and subsequent amendments 021, 0313, and 020). For this type of study, formal consent was not required.

## Results

### Evaluation study

In total, 267 blood culture broth samples from individual patients were assessed. Two samples of the *B. pseudomallei* panel tested negative with the InBiOS AMD RDT, and upon subculture of the broth and incubation for 48 h, no growth or growth with a different organism was seen. These samples were excluded from subsequent analysis, leaving 265 samples in the final collection: 114 samples with growth of *B. pseudomallei*, 139 samples with growth of other pathogens, and 12 samples with no growth (Table 1). The median storage time at − 80 °C for these samples was 18 months (interquartile range (IQR) 8–38 months). Of patients with *B. pseudomallei* infection, 62% was male and median age was 53 years (IQR 40–62). Of patients with infections other than *B. pseudomallei*, 42% was male and median age was 50 years (IQR 36–62). Overall, 43.8% (116/265) and 56.2% (149/265) of samples were collected from resin and charcoal bottles respectively.

**Table 1:** Number of samples included in the *B. pseudomallei* panel and the panel with competing isolates, and the results when performed on the InBiOS AMD RDT for melioidosis

Results of performance of InBiOS AMD RDT for melioidosis			
		Results	
Species in grown blood cultures	Samples *n* (% of total)	InBiOS AMD RDT positive *n* (%)	InBiOS AMD RDT negative *n* (%)
*Burkholderia pseudomallei* panel			
*Burkholderia pseudomallei*	114	110 (96.5)	4 (3.5)
Competing panel	139		
Non-fermentative Gram-negative organisms	19 (13.7)	0	19
*Acinetobacter* species	5	0	5
*Burkholderia cepacia*	5	0	5
*Pseudomonas* species	6	0	6
*Sphingobacterium* species	1	0	1
*Sphingomonas* species	2	0	2
Gram-negative bacilli	66 (47.5)	0	66
*Escherichia coli*	21	0	21
*Klebsiella oxytoca*	1	0	1
*Klebsiella ozaenae*	1	0	1
*Klebsiella pneumoniae*	12	0	12
*Salmonella* Choleraesuis	2	0	2
*Salmonella* Paratyphi A	6	0	6
*Salmonella* species	6	0	6
*Salmonella* Typhi	5	0	5
*Enterobacter* species	4	0	4
*Aeromonas* species	5	0	5
Unknown Gram-negative bacilli	1	0	1
*Haemophilus influenzae*	1	0	1
*Haemophilus parainfluenzae*	1	0	1
Gram-positive cocci	25 (18.0)	0	25
*Staphylococcus aureus*	12	0	12
*Streptococcus pneumoniae*	3	0	3
*Streptococcus* species	6	0	6
*Streptococcus suis*	1	0	1
*Enterococcus faecalis*	1	0	1
*Enterococcus* species	2	0	2
Probable contaminants	29 (20.9)	0	29
*Bacillus* species	11	0	11
*Corynebacterium* species	6	0	6
*Staphylococcus non-aureus*	12	0	12
No growth	12	0	12

### Sensitivity and specificity

There was a total agreement between the two observers on the test result in all cases, and no invalid test results were noted. Test sensitivity was 96.5% (95% CI: 91.3–98.6%) with 4/114 (3.5%) of *B. pseudomallei* samples that showed no test line and that were hence categorized as false-negative. The four false-negative samples were collected in 2010, 2012, 2016, and 2017. None of the samples of the competing panel, including the blood culture broth samples with no growth, showed a visible test line; specificity was 100% (95% CI: 97.5–100%). There was no difference in sensitivity between the charcoal and the resin bottles (97.5% CI: 90.3–99.6% versus 94.1% CI: 79.0–99.0%, respectively, *p* value = 0.582).

### Line intensities, background clearance, and delayed reading

Of 110 true-positive samples, the majority (96/110, 87.3%) of test lines had medium or strong line intensities and only 1.8% (*n* = 2/114) test lines were scored “faint” by both readers. Kappa values for line intensities were considered “good” with differences between the two readers limited to only one category of intensity (Kappa value 0.639, CI 95%: 51.4–76.3%). Of the 265 tests observed, the strip background was light in 59.2% of cases, medium in 34% of cases, and high in 6.8% of cases, but in any case, reading of the test lines was not hindered. Delayed reading did not reveal any appearing test lines after 1 h. No difference between samples collected in charcoal versus resin bottles was noted for sensitivity (*p* = 0.582), line intensities (*p* = 0.1611), or background clearance (*p* = 0.162).

### Repeatability

Two *B. pseudomallei* samples had false-negative results for both tests. From the 17 *B. pseudomallei* samples that showed positive results for both tests, 9 (53%) showed no difference in line intensity between the two tests, whereas difference occurred within one level of test line intensity in 7 (41%) other cases. The remaining sample showed two category differences in line intensities between the two tests (strong-weak). In addition, for one sample of the competing panel (*Enterococcus* species), one of the tests was negative but a faint line was observed for the other test.

### Assessment of the prozone effect

**Fig. 3:**
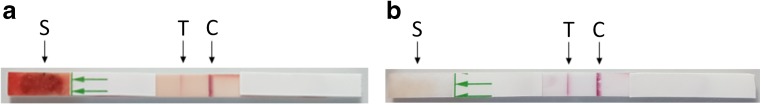
Example of the prozone effect in a sample with growth of *B. pseudomallei*. **a** Undiluted sample and **b** sample 10× diluted in NaCl 0.9%

A prozone effect was observed in one of the samples. The first performance of the test generated a weak test line whereas a strong test line intensity was recorded upon 10-fold dilution (Fig. 3a, b).

### Procedure performed with and without centrifugation step

Comparisons between line intensities and background clearance were available for 18 out of 19 samples. One sample showed a false-negative result. For the 18 samples with true-positive results, there was no difference in samples processed with and without centrifugation for line intensity (*p* = 0.167) and background clearance (*p* = 0.053). A tendency for stronger line intensities for the samples processed without centrifugation was observed: two weak test line intensities with centrifugation were observed as strong and medium line intensities without centrifugation.

### Test of the processed InBiOS strips for presence of viable bacteria

One third (6/19, 31.6%) of the *B. pseudomallei* positive test strips yielded growth of *B. pseudomallei* when incubated on MacConkey agar. There was no difference in growth on the MacConkey agar between centrifugated and non-centrifugated samples (*p* = 0.053).

### Evaluation of the instructions for use and ease of use

The IFU clearly explained the product, the principle of the RDT, the intended use, and the materials provided. It contained a version number and an effective date (19 September 2013) but not the product code. It also explained that the product is marketed for research only and that sensitivity and specificity of the test were not mentioned since they have not been formally established yet.

Materials required but not provided in the test box (e.g., test tubes, transfer pipette) were clearly indicated on the IFU.

Regarding ease of use of the InBiOS AMD RDT, it was noted that the test strip was not housed in a cassette and offered insufficient space to write the patient or sample identification. The expected place of appearance of control and test lines was not indicated (Fig. 2). In addition, although the IFU warned for damage to humidity, there was no visible desiccant added to the packaged strips.

The test procedure was described in detail in the IFU. For testing of direct blood samples, the IFU recommended to transfer the test strip into a tube preloaded by chase buffer. For testing of blood culture samples, there was no recommendation given. In our experience, the three drops of chase buffer, added after application of the sample suspension, easily overflowed the small nitrocellulose strip of the InBiOS AMD RDT which hampered the safe manipulation of the test strip and which increased the risk of spills. Further, the IFU warned for exposure to the chase buffer (containing an irritating preservative) but the buffer vial was not labeled accordingly. Storage and test procedure temperatures were mentioned to be 20–28 °C with referral to a potential impact on test results at temperatures above 30 °C.

## Discussion

The performance of the InBiOS AMD RDT for the detection of *B. pseudomallei* in stored grown blood culture broth showed a high accuracy (96.5 and 100% sensitivity and specificity respectively). When taking into account one false-positive result recorded with the assessment of repeatability, the specificity of the test was 99.3% (95% CI: 0.96–0.999). Reading of the test was straightforward with clearly visible test lines, good background clearance, and excellent inter-observer agreement. Performance was equally well from charcoal and resin BacT/Alert bottles. The procedure performed without centrifugation yielded similar results as the procedure with centrifugation. Repeatability was satisfactory, with 53% of samples showing no difference in line intensity between the first and the second reading. The IFU clearly indicated the test purpose and the test procedure.

To the best of our knowledge, the present study is the first to apply the InBiOS AMD RDT on blood culture broth. The excellent accuracy is in line with what is expected in light of previous testing performed on grown isolates by Houghton and coworkers [13]. In this study, analytical sensitivity was 98.7% when challenged with a large panel of *B. pseudomallei* isolates compiled by the US Stakeholder Panel on Agent Detection Assay. As both *B. pseudomallei* and *B. mallei* produce an identical mannose-heptose capsule, the InBiOS AMD RDT reacts equally well with *B. mallei* [25]. *B. mallei* causes glanders, an often fatal, zoonotic disease mainly affecting solipeds [26, 27]. Given the rare occurrence of human glanders and the antibiotic treatment similar to melioidosis [28], the co-reactivity of the InBiOS AMD RDT is not expected to be a problem in melioidosis-endemic settings. In non-endemic settings, this co-reactivity is an advantage as both pathogens are potential agents of bioterrorism [29].

The specificity in the current study matches the high analytical specificity obtained in competing isolates demonstrated in the aforementioned study [13]. The present study also demonstrated non-reactivity of the InBiOS AMD RDT with samples grown with *B. cepacia* and other Gram-negative rods, which may occur as healthcare-associated pathogens [30]. Of note, one case of prozone was observed. Given the high concentration of bacteria in grown blood (over 10^8^/ml [31]), prozone can be expected in antigen-detecting RDTs such as in the case of malaria [32]. Although rare and in this case displaying a weak but not negative test line, post-market evaluation and monitoring of this phenomenon should be conducted, and a referral in the IFU may be considered. The InBiOS AMD RDT showed similar results for the resin and the charcoal bottles, which is promising for the use of InBiOS AMD RDT on blood culture samples from different types or brands of blood culture bottles. In addition, the procedure performed without centrifugation provided similar results compared to the procedure with centrifugation. Since centrifugation of cultures grown with *B. pseudomallei* challenge biosafety because of possible aerosol formation, performing the procedure without the centrifugation step is preferred. The strip format can still be improved by addition of a cassette housing.

Although the limit of detection of the InBiOS AMD RDT is as low as 0.2 ng/ml making it suitable to detect *B. pseudomallei* in clinical samples [13], diagnostic sensitivity assessed on 40 direct blood samples was only 40% [33]. This may be explained by the low load of *B. pseudomallei* in blood (0.1–100 CFU/ml) [34]. To our knowledge, sensitivity of the InBiOS AMD RDT for other specimens like urine, sputum, or pus has not yet been published but will probably be higher, given the higher bacterial load compared to blood [13].

As to operational performance, there are some concerns about biosafety. First, the centrifugation step required for testing on blood culture broths and, according to the IFU, other culture fluids, challenges biosafety as airtight centrifuge buckets are needed to reduce the risk of aerosol formation. We demonstrated equal performance in terms of line intensity and background clearance of the InBiOS AMD RDT in non-centrifuged blood broth samples with growth of *B. pseudomallei*. If confirmed on larger sample sizes and on different blood culture brands, the procedure may be adapted accordingly. Further, about one third of processed InBiOS AMD RDT strips still yielded viable bacteria, which is of particular concern since adding the chase buffer to the nitrocellulose strip resulted in spill. Improvement of the design (e.g., replacement of the tubes by closed vials with a dropper, embedding the nitrocellulose strip in a cassette) may improve both biosafety and ergonomics, particularly in diagnostic laboratories with minimal equipment. Further, the InBiOS AMD RDT can be optimized by provision of more space to write sample identification, the addition of a desiccant with humidity indicator, improved labeling and stability testing of the test strip in tropical (> 30 °C) temperatures.

In low-resource settings, melioidosis has a mortality rate of up to 53% [6, 11]. This high mortality rate is associated with a delay in diagnosis, as conventional empiric antibiotic regimens do not provide adequate coverage for *Burkholderia pseudomallei* [1]. Identification of the grown isolate in the laboratory is challenging and can take up to several days [12]. In addition, misidentifications are not uncommon in laboratories that are not used to the organism [35, 36]. The present study showed that, when applied to grown blood cultures, the InBiOS AMD RDT has the potential to shorten the identification step. In case of the laboratory work-up at SHCH, this reduction in time to identification would be 24 h (preliminary identification) to 48 h (confirmed identification). One alternative shortcut in identification is the latex agglutination that detects a 200 kD component surface antigen of arabinose negative *B. pseudomallei* (Mahidol University, Thailand [19]). This latex test has shown similar sensitivity and specificity on grown blood culture fluid of BacT/Alert bottles (sensitivity and specificity respectively 95.8 and 100%) as the InBiOS test. The latex test is however not commercially available and has a cold-chain requirement. Another shortcut for the identification of *B. pseudomallei* is the detection of serum antibodies to *B. pseudomallei*, but most clinicians and researchers refer to the limited value of indirect hemagglutination assay (IHA), a commonly used serological diagnostic test in clinical care, because of the inconsistent appearance of antibodies and the high background seropositivity rate in endemic populations [7, 37, 38]. A recent publication, however, showed that serological detection and monitoring of melioidosis disease in endemic areas is possible, but a high cut-off threshold needed to be set on the developed ELISA tests. An accurate, rapid point of care test for serological diagnosis has not yet been developed [39].

Given its high accuracy and speed, the InBiOS AMD RDT represents a promising tool to reduce the diagnostic delay of melioidosis in endemic settings. A next step is defining the optimal way of integrating the InBiOS AMD RDT in the work-up of grown blood cultures in low-resource settings, validating other types and brands of blood culture media for use with the InBiOS AMD RDT and assessing its operational performance and adoption in a prospective study.

Among the limitations of the study was firstly the duration of storage of samples for a period up to 7 years. Influence of this storage time on the test results is however unlikely since the collection dates of the false-negative samples cover the whole collection range (2010–2017). Secondly, the collection of samples was done in a busy diagnostic laboratory and not in a dedicated research setting. As such, clerical errors or contamination of samples during storage might be expected, as was potentially the case for the two samples that were excluded from further analysis. Likewise, the freeze-thawing procedure may have lysed some remaining red blood cells, resulting in a better migration and background clearing compared to fresh blood culture broth samples.

Apart from these limitations, this study has several strengths. The performance of the *B. pseudomallei* detecting RDT was assessed on a panel of blood culture broth samples that presented the key pathogens of the endemic setting and included non-fermenting Gram-negative rods close to the *Burkholderia* genus. In addition, retrospective testing allowed the inclusion of a large number of samples resulting in narrow confidence intervals of the test characteristics.

## Conclusion

In this study, we demonstrated excellent accuracy for the InBiOS AMD RDT for the detection of *B. pseudomallei* antigen in stored blood culture broth. Provided further adaptations, the InBiOS AMD RDT is a valuable tool to be integrated in the work-up of blood cultures in low-resource settings.

## Electronic supplementary material

ESM 1 Dataset Evaluation InBiOS AMD Rapid Diagnostic Test (XLSX 72 kb)

ESM 2 Analysis Instructions for use (IFU) (XLSX 10 kb)

ESM 3 STARD checklist (DOCX 30 kb)
